# Liquid Hot Water Pretreatment and Enzymatic Hydrolysis as a Valorization Route of Italian Green Pepper Waste to Delivery Free Sugars

**DOI:** 10.3390/foods9111640

**Published:** 2020-11-10

**Authors:** M.A. Martín-Lara, L. Chica-Redecillas, A. Pérez, G. Blázquez, G. Garcia-Garcia, M. Calero

**Affiliations:** 1 Chemical Engineering Department, Faculty of Sciences, University of Granada, Avda. Fuentenueva, s/n, 18071 Granada, Spain; luciachica@correo.ugr.es (L.C.-R.); aperezm@ugr.es (A.P.); gblazque@ugr.es (G.B.); mcaleroh@ugr.es (M.C.); 2 Department of Chemical and Biological Engineering, The University of Sheffield, Sir Robert Hadfield Building, Sheffield S1 3JD, UK; G.Garcia-Garcia@sheffield.ac.uk

**Keywords:** pretreatment, hydrolysis, Italian green pepper, liquid hot water, fermentable sugars, glucose, xylose, food waste

## Abstract

In this work, liquid hot water pretreatment (autohydrolysis) was used to improve enzymatic hydrolysis of a commonly consumed vegetable waste in Spain, Italian green pepper, to finally produce fermentable sugars. Firstly, the effect of temperature and contact time on sugar recovery during pretreatment (in insoluble solid and liquid fraction) was studied in detail. Then, enzymatic hydrolysis using commercial cellulase was performed with the insoluble solid resulting from pretreatment. The objective was to compare results with and without pretreatment. The results showed that the pretreatment step was effective to facilitate the sugars release in enzymatic hydrolysis, increasing the global sugar yield. This was especially notable when pretreatment was carried out at 180 °C for 40 min for glucose yields. In these conditions a global glucose yield of 61.02% was obtained. In addition, very low concentrations of phenolic compounds (ranging from 69.12 to 82.24 mg/L) were found in the liquid fraction from enzymatic hydrolysis, decreasing the possibility of fermentation inhibition produced by these components. Results showed that Italian green pepper is an interesting feedstock to obtain free sugars and prevent the enormous quantity of this food waste discarded annually.

## 1. Introduction

About one-third of the total production of food in the world is wasted, amounting to 1300 million tons of food waste globally in 2011 [1]. In developing countries, food waste occurs mainly during cultivation, harvesting, transportation, and storage due to the use of inadequate and rudimentary techniques and technologies. On the contrary, in medium- and high-income countries food waste occurs mainly in the last stage of the food supply chain, i.e., households and food service establishments. Nevertheless, in medium- and high-income countries, cultivation, harvesting, and preserving techniques also produce significant losses of food, mainly due to strict quality regulations, such as limitations in size, shape, and color [1].

Fruits and fresh vegetables have one of the highest waste rates; approximately half of the total production of fruit and fresh vegetables is wasted [2,3]. Most of this waste is generated in Europe, since 49.4% of holdings that grow fresh vegetables that are consumed within in the European Union (EU) are located in Romania (22.1%), Poland (15.4%), and Spain (11.9%) [4]. This EU report also indicated that almost 2.2 million hectares of land are used to produce vegetables for fresh consumption or further processing. About 17% of the EU lands used to produce fresh vegetables are located in Spain, which mainly produce the following vegetable crops: zucchini, cucumbers, eggplants, gherkins, melons, peppers, and tomatoes (27.6% of the Spanish produce) [4].

In this context, pepper (*Capsicum annuum*) is one of the most valuable vegetables [5]. Statista Research Department reported that about 36 million tons of peppers were produced in the world in 2018 [6]. China is the world’s leading producer with around half of the peppers produced globally. Spain ranks fifth in the world’s pepper production with 1.3 million tons [7]. With their 18,513 hectares of land for pepper production and a yield of 6.11 kg/m^2^, Spain has the highest yield of the ten largest producers in the world [7,8,9,10,11]. Spain is also the largest European producer of pepper. The Netherlands is the second largest European producer, with a much lower production than Spain (0.34 million tons), followed by Italy [12]. Since the start of the pepper boom, between 40 to 45 years ago, Spanish production has continuously grown, with a current increase rate of around 5% every year [12]. 

Pepper waste represents a considerable percentage of the total vegetable waste generated in Spain [12]. Furthermore, due to its high content of carbohydrates (75.9% in dry weight according to data from USDA), pepper waste has promising potential for the extraction of fermentable sugars. The potential production of bioethanol from these waste materials needs to be carried out by hydrolysis because some of the carbohydrates are in polymeric form (such as, cellulose and hemicellulose). The pretreatment of the pepper waste to facilitate the polysaccharides hydrolysis is a key step in the biotechnological procedure of pepper waste valorization [13].

Previous research has shown the performance of different biological, chemical, physical, and physicochemical pretreatments in order to transform the structure of cellulose, hemicellulose, and lignin and facilitate the depolymerization and liberation of fermentable sugars. For example, Cabrera et al. [14] explored alkaline and alkaline peroxide pretreatments at a mild temperature to enhance the enzymatic hydrolysis of rice hulls and straw. Hassan et al. [15] reviewed the pretreatment of lignocellulosic materials by emerging methods such as ultrasound and ionizing radiation. Intanakul et al. [16] revealed the advantages of microwave pretreatment to improve enzymatic hydrolysis of rice straw and sugarcane bagasse. Similarly, Loow et al. [17] revised the use of deep eutectic solvents and highlighted the advantage of using them as pretreatment agents of lignocellulosic materials. Sarkar et al. [18] and Sun and Cheng [19] summarized the main physical, chemical, physicochemical, and biological pretreatment techniques to facilitate the enzymatic attack in their review about available technologies for bioethanol production from lignocellulosic materials. Talebnia et al. [20] provided an overview of pretreatment technologies investigated for wheat straw before its transformation to bioethanol. The pretreatment technologies analyzed included physical (i.e., milling), physico-chemical (i.e., liquid hot water), chemical (i.e., dilute acids) and biological technologies (i.e., fungal treatments). Wan et al. [21] compared liquid hot water and alkaline pretreatment of soybean straw and showed that liquid hot water pretreatment was better than alkaline pretreatment. A combination of treatments has also been explored to see if a mixture of treatment can yield better results [22]. After this step, lignocelullosic materials became susceptible to hydrolysis and yields of monomeric sugars were increased [23]. The comprehensive overview of existing pretreatment technologies undertaken by Arshadi et al. [24], focused on wastes throughout the agri-food chain, was also of special interest. As a conclusion, the pretreatment is a key step in the processing of food waste. This process consists of the following steps: (1) pretreatment, (2) an enzymatic hydrolysis using commercial enzymes to release fermentable sugars, and (3) fermentation of the resulting sugar solution.

In studies focused on fresh vegetables, the hydrothermal pretreatment with dilute acids has been tested in more detail [13,25,26]. However, the development of greener pretreatments that avoid the use of chemicals is essential. In this context, novel high-pressure CO_2_–H_2_O processes, to produce oligosaccharides from the hemicellulose fraction and to enhance the cellulose digestibility for the enzymatic hydrolysis, is increasing the attention of researchers [27,28,29,30]. Another interesting green pretreatment is liquid hot water (autohydrolysis), commonly known as “green pretreatment process”. It is a chemical-free procedure that does not require the addition of a catalyst. This pretreatment relies on pressure to maintain water in the liquid state at elevated temperatures [31,32,33,34,35,36,37,38,39]. Another benefit of the liquid hot water pretreatment (autohydrolysis) is that the formation of degradation products as phenolic compounds, furans, aliphatic carboxylic acids or uronic acids is low [40]. The disadvantage of this method, according to Manzanares et al. [36], is that liquid hot water pretreatment has high water and energy requirements. In any case, this pretreatment is receiving attention to be developed at the commercial scale.

Although optimization of liquid hot water pretreatment technology was previously reported in work by Wells et al. [41], the success of the “auto-hydrolysis process” depends mainly on crop type, harvest year, location, etc. and it needs to be carefully optimized for maximizing the final result. To the best of our knowledge, this pretreatment with hot water has scarcely been studied for green pepper. Only Díaz et al. [13] has published a study that describe the hydrolysis of supermarket vegetable wastes that included green pepper as substrate and some tests of the thermal hydrolysis performance. 

In this context, the aims of the work presented in this article were as follows: (1) to investigate the efficiency of hydrothermal pretreatment as a technology for Italian green pepper waste pretreatment, (2) to optimize the pretreatment conditions in terms of temperature and operation time to maximize both hemicellulosic and cellulosic sugar recovery, and (3) to evaluate the enhancement of the sugar recovery as well as the minimization of inhibitors (phenolic compounds) in the liquid fraction of the enzymatic hydrolysis. This work supports the use of vegetable waste as raw materials to produce fermentable sugars.

## 2. Materials and Methods 

### 2.1. Raw Material: Italian Green Pepper Waste

The Italian green pepper waste used in this study was collected from a greenhouse crop residues treatment plant. The industrial plant covers a space of more than 24 hectares, and is located at “Cortijo Galindo”, in the municipality of Motril (Granada, Spain). It treats vegetable waste generated in the area, especially from intensive greenhouse agriculture. Peppers (*Capsicum annuum* L.) were selected as raw material of this study because they are one of the most important greenhouse crops produced in Granada. The material investigated in this work was the greenhouse crop residue from green peppers cultivation, which mainly contained the vegetable itself, but also prunings and other woody plants that could be present in greenhouse residues.

The material samples were firstly washed with distilled water, cut to a size of approximately 5 mm and then dried at 50 °C. The resultant solid material was ground to a particle size lower than 2 mm to optimize surface area prior to pretreatment. Finally, the material was stored at 10 °C until further use. The raw material mentioned below refers to the green pepper sample before pretreatment, denoted as RGP (raw green pepper). 

### 2.2. Characterization of Italian Green Pepper Residue Samples

A structural analysis of the samples was performed by ASTM D1106-96:2013 method (lignin) [42], chlorination method (holocellulose) [43], ASTM D1103-60:1977 (cellulose) [44], and ASTM D1107-96:2013 (extractives) [45]. Hemicellulose content was calculated by subtracting the content of holocellulose from the content of cellulose. The carbohydrate, protein, and lipid contents were obtained from the food composition databases from the United States Department of Agriculture (USDA).

The total moisture content was determined by measuring the weight of the wet sample (as received) and the weight of the same sample after drying by air in a drying oven (Raypa, DOD50) at 50 °C until constant weight (approximately 72 h).

The proximate analysis was determined according to the following international standardized methods of analysis of solid biofuels of the International Organization for Standardization (ISO): ISO 18123:2015 (volatile matter) [46], ISO 18122:2015 (ash content) [47], and ISO 18134-1:2015 (equilibrium moisture content) [48]. Finally, fixed carbon content was calculated by difference (volatile matter, ash content, equilibrium moisture content, and fixed carbon must sum 100%).

Determination of elemental composition (carbon, hydrogen, nitrogen, sulfur and oxygen concentrations) was performed using a Fisons, EA 1108 CHNS, elemental analyzer (Thermo Fisher Scientific, Madrid, Spain).

To experimentally determine the potential sugars, including glucose and xylose, 0.5 g of sample free of extracts and dried was introduced into an Erlenmeyer flask to which 5 mL of 72% H_2_SO_4_ was added, and then kept in a water batch at 30 °C for one hour making periodic agitations. Next, the solution was diluted by adding distilled water to reach 4% H_2_SO_4_. Then, the dilute solution was then autoclaved at 121 °C for one hour. Finally, the solution was cooled down to room temperature, and filtered on a pore plate number 3, obtaining a solid phase for quantification of the lignin and a liquid phase that was frozen until later analysis. Specifically, potential sugars were determined by high performance liquid chromatography (HPCL), according to analytical methods described in Section 2.5.

The bulk density of dried and milled sample was determined following standard EN 15103, using a standardized container.

Finally, infrared analysis was performed in the range of 4000–400 cm^−1^ using a Fourier transformed infrared spectrophotometer (FTIR), Perkin-Elmer, Spectrum-65 model (Perkin-Elmer, Madrid, Spain).

### 2.3. Liquid Hot Water Pretreatment

Experiments were performed with 50 g of green pepper samples that were mixed with 500 mL distilled water (weight/volume ratio of 10%). The suspension was heated in a high pressure, bolted closure laboratory stirred reactor (ILSHIN) at 150, 165, and 180 °C with pressure maintained at 4.8, 7.5, and 10.8 bar, respectively. After reaching the operating temperature, the suspension was held at a stable temperature for 10 and 40 min. Then, the solids were separated with a sieve with a size of 125 µm and the liquid phase was frozen at −20 °C until being analyzed. The insoluble solid (IS) residue was dried and stored at 10 °C until further use.

The operating conditions were chosen according to recent published works on the use of liquid hot water techniques as pretreatment of lignocelullosic materials for the production of fermentable sugars [41,49].

### 2.4. Enzymatic Hydrolysis (Saccharification Step)

The protocol of the enzymatic hydrolysis was based on studies of Binod et al. [50], Larnaudie et al. [51] and Manzanares et al. [36]. Enzymatic hydrolysis was carried out in glass bottles of a total volumetric capacity of 100 mL by mixing 8 g of RGP or IS resulting from pretreatment with 80 mL of distilled water. Commercial enzyme from Trichoderma reesei, Celluclast^®^ 1.5 L (aqueous solution, enzymatic activity ≥700 Endoglucanase units/g, density 1.10–1.30 g/mL; provided by Sigma-Aldrich, (Madrid, Spain) was added to suspension in a charge of 15 units by gram of solid. For this operation, the pH was adjusted to 4.5 and the mixture was treated for 24 h at 50 °C (optimal temperature for celluclast) at a stirring rate of 300 rpm. Afterwards, the bottles were treated to 95 °C for 5 min in order to stop the enzymatic reaction. Finally, samples were sieved, centrifuged and filtrated to separate solid and liquid fractions. The liquid fraction was frozen at −20 °C until being analyzed. 

### 2.5. Analytical Methods

The total phenols of the liquid fractions obtained from the enzymatic hydrolysis were measured using the Folin–Ciocalteu method, as reported by Greenberg et al. [52], with gallic acid as reference substance. Folin–Cicolteau, sodium carbonate, and gallic acid reagents were provided by Sigma-Aldrich. After the method was applied, a reaction based on electron transfer was produced and a blue color, proportional to the number of hydroxyl groups, was formed in samples. Then, samples were measured at 765 nm in a UV–visible spectrophotometer (Thermo Spectronic, Genesys 6, Madrid, Spain).

The glucose and xylose contents of all the liquid fractions were determined using high performance liquid chromatography (HPLC). Among the multiple types of HPLC that exist, we chose ion chromatography because of its widespread use to separate monosaccharides. Particularly, the 940 Professional IC Vario equipment (Methrohm, Herisau, Switzerland) was used. Glucose and xylose were used as monosaccharide standards. A column MetrosepCarb 2 250/4.0 was used under the following conditions: mobile phase, 100 mM NaOH and 10 mM NaAc; flow rate, 0.5 mL/s; column temperature, 303 K, which were the same operating conditions as those reported in our previous work [53].

### 2.6. Parameters Definition

In order to evaluate the effects of different pretreatment conditions, a series of parameters was used that referred to the changes in the raw material as a result of the pretreatment. Equations for the determination of these parameters are described below. Solid materials in the different equations are considered on a dried basis as follows.

- Solid yield (SY, %)

This parameter gives information about the amount of raw material solubilized as a consequence of the effect of the pretreatment. It is defined as the amount of solid material obtained after the pretreatment stage (insoluble solid (IS) or pretreated material), in relation to the amount of raw material introduced (raw green pepper (RGP)), using Equation (1):

(1) $$\mathrm{SY},\;\%=\frac{g\;\mathrm{of}\;\mathrm{IS}}{g\;\mathrm{of}\;\mathrm{RGP}}\times 100$$

- Component concentration in the pretreated material

The concentration of each component (glucose and xylose) in the pretreated material is calculated based on the amount of this component in the pretreated material referred to as the dry weight of the pretreated material:

(2) $$\mathrm{Component}\;\mathrm{concentration},\;\%=\frac{g\;\mathrm{of}\;\mathrm{component}\;\mathrm{in}\;\mathrm{IS}}{100\;g\;\mathrm{of}\;\mathrm{IS}}$$

- Recovery of components after pretreatment

This variable refers to the amount of each component in the fraction considered (insoluble solid (IS) or liquid fraction (LF)) based on its content in the raw material. It provides information on the recovery of each component after the pretreatment stage:

(3) $$\mathrm{Component}\;\mathrm{recovery}\;\mathrm{IS},\;\%=\frac{g\;\mathrm{of}\;\mathrm{component}\;\mathrm{in}\;\mathrm{IS}\;\mathrm{by}\;\mathrm{gram}\;\mathrm{of}\;\mathrm{IS}}{g\;\mathrm{of}\;\mathrm{component}\;\mathrm{in}\;\mathrm{RGP}\;\mathrm{by}\;\mathrm{gram}\;\mathrm{of}\;\mathrm{RGP}}\times \mathrm{SY}$$

(4) $$\mathrm{Component}\;\mathrm{recovery}\;\mathrm{LF},\;\%=\frac{g\;\mathrm{of}\;\mathrm{component}\;\mathrm{in}\;\mathrm{LF}\;\mathrm{from}\;\mathrm{pre}-\mathrm{treatment}}{g\;\mathrm{of}\;\mathrm{component}\;\mathrm{in}\;\mathrm{the}\;\mathrm{amount}\;\mathrm{of}\;\mathrm{RGP}\;\mathrm{used}\;\mathrm{in}\;\mathrm{pre}-\mathrm{treatment}}\times 100$$

- Saccharification efficiency (SE, %)

This parameter shows the amount of component released during the enzymatic hydrolysis (EH) step, in relation to the amount of potential component in the pretreated material or in the raw material. It provides information about the susceptibility of the IS to the enzymatic attack result, and as such, it is an important variable to measure the effectiveness of the pretreatment:

(5) $$\mathrm{SE},\;\%=\frac{g\;\mathrm{of}\;\mathrm{component}\;\mathrm{in}\;\mathrm{LF}\;\mathrm{from}\;\mathrm{EH}}{g\;\mathrm{of}\;\mathrm{component}\;\mathrm{in}\;\mathrm{the}\;\mathrm{amount}\;\mathrm{of}\;\mathrm{IS}\;\mathrm{used}\;\mathrm{in}\;\mathrm{EH}}\times 100$$

- Global efficiency (GE, %)

This variable refers to the amount of component in the raw material capable of being fermented after the stages of pretreatment and enzymatic hydrolysis. It is calculated as the summation of the component content in the liquid fraction (LF) and the one generated in the EH, referring to the content of component in the raw material:

(6) $$\mathrm{GE},\;\%=\frac{\frac{g\;\mathrm{of}\;\mathrm{component}\;\mathrm{in}\;\mathrm{LF}\;\mathrm{from}\;\mathrm{EH}}{g\;\mathrm{of}\;\mathrm{IS}}\times \frac{\mathrm{SY}}{100}+\frac{g\;\mathrm{of}\;\mathrm{component}\;\mathrm{in}\;\mathrm{LF}\;\mathrm{from}\;\mathrm{Pre}-\mathrm{treatment}}{g\;\mathrm{of}\;\mathrm{RGP}}}{g\;\mathrm{of}\;\mathrm{component}\;\mathrm{in}\;\mathrm{RGP}\;\mathrm{by}\;\mathrm{gram}\;\mathrm{of}\;\mathrm{RGP}}\times 100$$

### 2.7. Statistical Analysis

All measurements were undertaken at least in triplicate. Data are presented as means with error bar and ± indicating standard deviations. Collected data were statistically analyzed using analysis of variance (ANOVA) and significant differences between means of data were compared. A post hoc analysis to determine which means are significantly different from which others was also implemented. Although there are several multiple comparison procedures, the Fisher’s least significant difference (LSD) procedure was chosen in this work.

## 3. Results and Discussion

### 3.1. Green Pepper Waste Characterization

The raw material used in this research was characterized according to the methods of analysis described in Section 2.2. It is important to highlight that our sample is the greenhouse crop residue from green peppers cultivation, which mainly contains the vegetable itself, but also prunings and other woody plants that could be in greenhouse residues. The raw material had a moisture content of 95.6%.

Table 1 shows the main composition of green pepper (% dry basis). The values are an average of three repetitions, except data of carbohydrates content that were adapted from USDA. Carbohydrates make up approximately 75.9% of the initial dry material, which makes this residue a very suitable substrate for the production of ethanol [54]. Its degree of lignification is in the range of what is described for other similar vegetable waste such as eggplant (7.47%) [55] or tomato (5.85%) [56]. The high ash content of Italian green pepper (7.0%) is also in agreement with results of other researchers. For example, a study about applications of pea peel found an ash content value for this solid of 5.65% [57]. Singh et al. [58] in their study about utilization of vegetable wastes for bioenergy generation also reported values between 6% and12% for potato samples. However, other authors, such as Pinna-Hernández et al. [59], recently found higher ash content (13.6%) for vegetable residues from greenhouse peppers.

With respect to elemental analysis, green pepper is mainly composed of carbon (43.9%) and oxygen (39.4%), with 6.4% of hydrogen, 3.2% of nitrogen, and practically no sulfur. According to our results, the sum of potential sugars reached approximately 50.5% of dry weight. This is in agreement with United Stated Department of Agriculture (USDA) data and with studies by Guil-Guerrero et al. [60] and Diaz et al. [13]. Regarding potential sugars, 66.7% of them were identified as glucose and 22.6% as xylose, while the sum of fructose, lactose, and saccharose represented only 10.7%. If the results of cellulose content are compared to potential glucose data, it can be concluded that the material has moderate non-cellulosic sugar content. This glucose is more likely to be monomeric glucose.

Finally, bulk density is an important parameter because it allows estimating storage and transport space needs and affects the handling and processing systems [61]. The bulk density value obtained for dried and milled Italian pepper was 431 kg/m^3^. This is a good value if it is compared with other feedstock materials such as those reported by Vasco-Correa and Shah [62]. It implies the need of equipment with less capacity to pretreat the same amount of material.

### 3.2. Performance of Hydrothermal Pretreatment

The evaluation of the performance of hydrothermal pretreatment was made analyzing the effect of temperature and contact time on the recovery of sugars in the solid and liquid fraction after pretreatment. 

#### 3.2.1. Solid Yield

**Figure 1:**
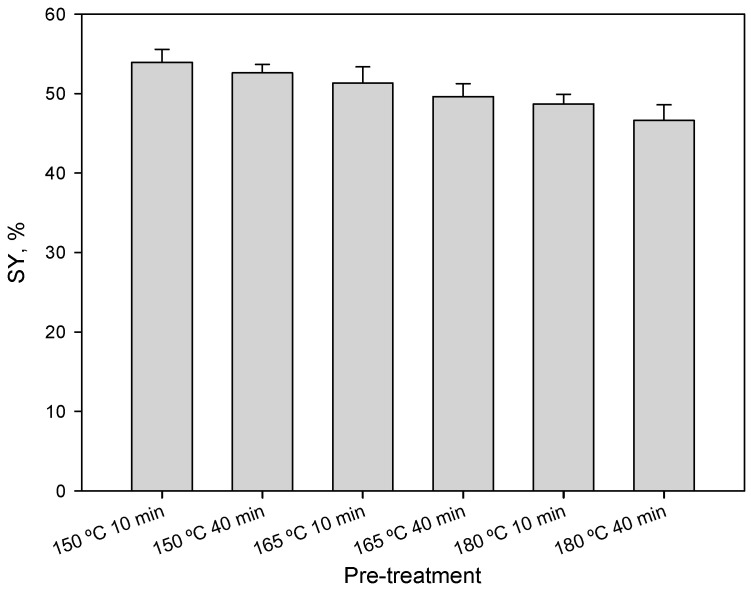
Solid yields (SY, %) for each operating conditions of liquid hot water pretreatment.

The solid yields (SY, %) for different temperatures and pretreatment times are shown in Figure 1. The values decreased from 53.9% to 46.6% as temperature and time increased, mainly due to the progressive solubilization of some components of the raw material such as hemicellulosic sugars, extractives, and soluble ashes. These are the compounds that are most easily solubilized in this type of hydrothermal pretreatment, since their structure is less complex than that of other components of the vegetable waste [41,49,51,63,64,65]. The decrease in the solid recovery was well in agreement with the literature [35,66,67,68,69]. Additionally, the low values (always lower than 55%) may be explained by the easy solubilization and volatilization phenomena of certain compounds, as previously described by Mok and Antal [64].

#### 3.2.2. Recovery of Sugars

**Figure 2:**
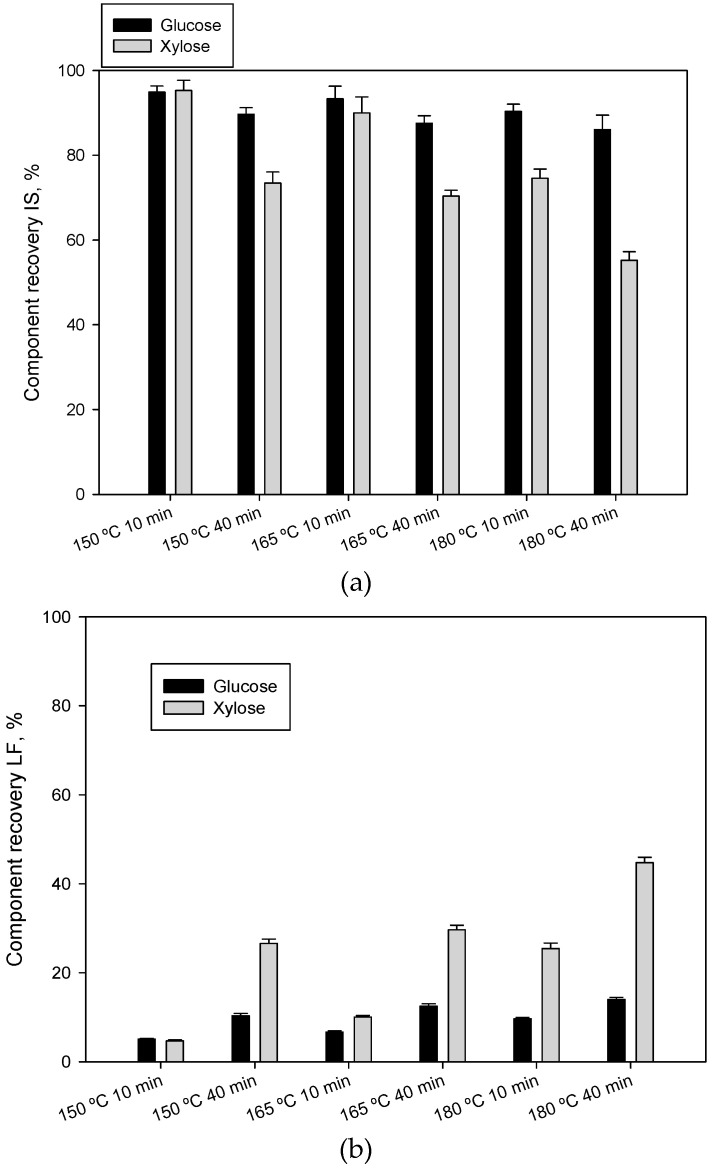
Sugar recovery for each operating conditions of liquid hot water pretreatment. (**a**) On insoluble solid (IS); (**b**) on liquid fraction (LF). Data were calculated for total potential glucose and xylose.

The recovery of sugars in the pretreated material (insoluble solid) and liquid fraction at different pretreatment conditions are shown in Figure 2a,b, respectively. Data were calculated for total potential glucose and xylose derived from cellulose and hemicellulose. The results refer only to glucose and xylose because they were the main sugars detected. Values of over 86% recovery of glucose in the insoluble solid were obtained. These results seem to indicate that operating conditions analyzed could result in a certain solubilization of the cellulose of the raw material. According to the data obtained, an increase in temperature and time produced a negative effect on glucose recovery in the insoluble solid. Regarding the recovery of xylose in the insoluble solid, Figure 2a shows the obtained results. It can be observed that temperature and time both have a greater effect on its release from the raw material, decreasing its content in the insoluble solid as the severity of the pretreatment is increased. The values decreased from 95.3% at operating conditions of 150 °C and 10 min to the lowest value of 55.24% at severe operating conditions of 180 °C and 40 min. 

Figure 2b shows the sugar recovery values in the liquid fraction. Glucose released as a consequence of the hydrothermal pretreatment was lower than 14% in all the experimental conditions, showing an important effect of the increase in temperature and time. On the one hand, the fact of finding glucose in the liquid fraction, even under conditions of smoother pretreatments, suggests that glucose could come from another source more easily soluble than cellulose. On the other hand, glucose could constitute an important sugar of the hemicellulosic component and could be released from this component [70]. In addition, the fact that the values are different under diverse operating conditions supports this hypothesis.

Temperature and time showed a stronger effect on the recovery values of xylose in the liquid fraction. At a high temperature (180 °C), the time increase from 10 to 40 min resulted in an increased xylose recovery from 25.42% to 44.76% (i.e., the maximum recovery value of xylose in the liquid fraction at the studied operating conditions). Similarly, when 40 min of operation time was chosen, a temperature increase from 150 to 180 °C caused an increase in the value of xylose recovery to 68.46%. Results also showed that pretreatment at 150 °C and 165 °C would require longer residence time (high than 40 min) if recoveries of xylose higher than to 30% were being sought. It could be hypothesized that a pretreatment conducted at a temperature greater than 180 °C and with a residence time longer than 40 min could lead to a higher recovery of xylose. However, in this case, some additional experimentation would be performed in order to analyze the degradation of these sugars under severe operating conditions. It is important to highlight that it is impossible to have zero sugar degradation at the analyzed operating temperatures when sugar release comes from soluble sugars (glucose and xylose) [70,71,72]. Xylose is especially sensitive. Therefore, some soluble sugars may be “lost” during pretreatment.

#### 3.2.3. Content of Structural Components in Residual Solids

With regard to structural components (cellulose, hemicellulose and lignin), the hydrothermal pretreatment using liquid hot water is capable of producing structural changes of the components of Italian green pepper waste. Without pretreatment, the green pepper waste is composed of 8.7% lignin, 24.8% cellulose and 10.7% hemicellulose, on a dry matter basis. Liquid hot water pretreatment resulted in a significant dissolution of hemicellulose. According to our results, hemicellulose was almost completely solubilized and deconstructed from biomass in hot water pretreatment at 180 °C for 40 min. These results agreed with data reported by Nitsos et al. [73] in their study about optimization of hydrothermal pretreatment of hardwood and softwood lignocellulosic residues for selective hemicellulose recovery and improved cellulose enzymatic hydrolysis. Additionally, Gullón et al. [74] reported that hemicellulose was the easiest fraction to hydrolyze and that the acetic acids released from the hemicellulose acetyl group hydrolysis were those that catalyze the material pre-treatment. On the contrary, cellulose and lignin were mostly preserved in the solid residues after liquid hot water pretreatment. The content of cellulose increased from 24.8% to 26.5% (*w*/*w*, dry matter). This could corroborate that cellulose was not especially altered during liquid hot water pretreatment. This is the reason why the enzymatic hydrolysis assays were performed using a fixed amount of enzyme (cellulase) per unit of solid. The approximately constant cellulose content of the analyzed samples explains the results reported in Section 3.3. Finally, the lignin content was slightly increased from 8.7% (*w*/*w*, dry matter) in the untreated Italian green pepper to about 10.1% (*w*/*w*, dry matter) in the pretreated solids. Similar results about cellulose and lignin contents were reported by Li et al. [35], Manzanares et al. [36], and Tian et al. [38] in their studies about the effect of liquid hot water pretreatment on the chemical-structural alteration of poplar, olive oil pomace residue and rigid hardwood, respectively.

**Figure 3:**
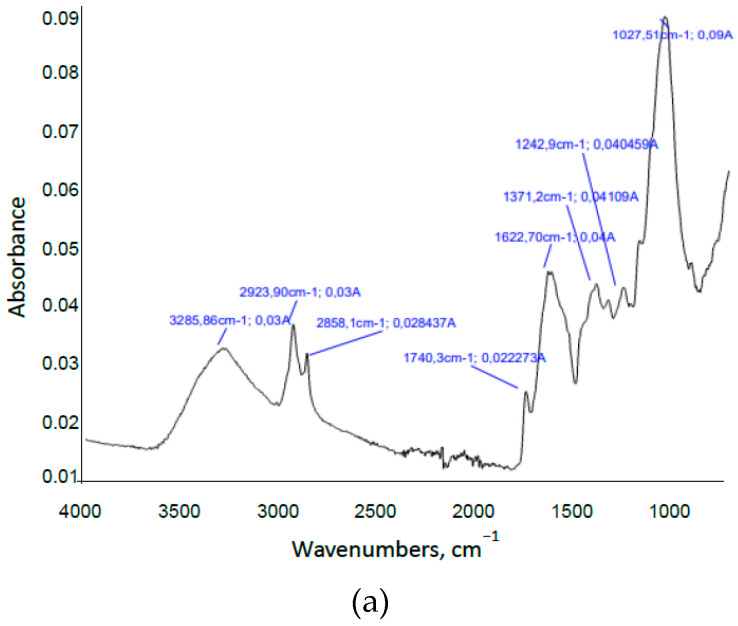

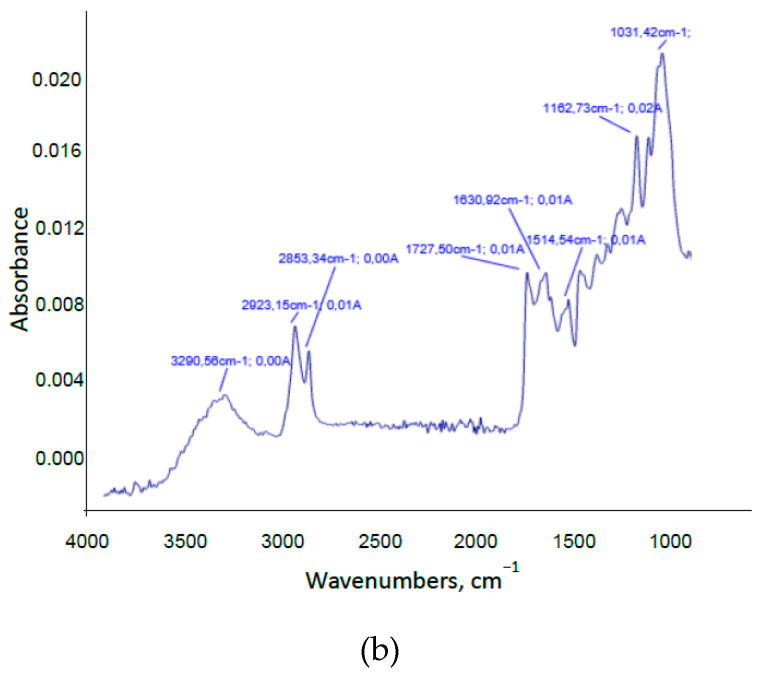
(**a**) FTIR spectra of raw material; (**b**) Insoluble solid resulting from liquid hot water pretreatment performed at 180 °C during 40 min.

The Fourier transform infrared spectroscopy (FTIR) technique was used to elucidate structural changes on the material during liquid hot water pretreatment. Figure 3a,b shows the infrared spectra of raw material and insoluble solid resulting from pretreatment at 180 °C for 40 min, respectively. The complexity and heterogeneity of materials were shown by the abundant and overlapped absorption bands. Representative bands/peaks found in spectrum of raw material were as follows [75,76]: 3286 cm^−1^ (O-H stretching), 2924 cm^−1^ (C-H stretching), 2858 cm^−1^ (-OCH_3_ methoxyl and C-H stretching), 1740 cm^−1^ (C=O stretching), 1623 cm^−1^ (C=C benzene stretching ring and C=O stretching conjugate with aromatic rings), 1371 cm^−1^ (symmetric C-H bending from methoxyl group), 1243 cm^−1^ (weak O-C stretching), and 1028 cm^−1^ (C-O ether vibrations and C-C ring vibrational stretching). After liquid hot water pretreatment, a decrease in absorbance was produced. An absorption peak at 1728 cm^−1^ attributed to C=O vibration of acetyl or COOH groups was also significantly enhanced (in relation to the rest of peaks/bands). A major number of small peaks between 1728 and 1031 cm^−1^ were found (characteristic of pure lignin spectra). Of special relevance were the new peaks found at wavenumber of 1514 cm^−1^, characteristic of aromatic skeletal vibrations of lignin, and 1163 cm^−1^, attributed to C-O-C asymmetric valence vibration. Finally, the characteristic peak at 1371 cm^−1^ of symmetric C-H bending from methoxyl group decreased as a consequence of the degradation of cellulose and hemicellulose. According to these results, a major proportion of lignin in the pretreated material seemed to be observed since lignin offered a characteristic peak at about 1514 cm^−1^, corresponding to the aromatic skeletal vibration, and a higher number of small peaks between 1750 and 1000 cm^−1^ approximately [73]. This observation could indicate that no depolymerization of lignin during the pretreatment occurred, as already suggested by Ázar et al. [77].

### 3.3. Performance of the Enzymatic Hydrolysis

**Figure 4:**
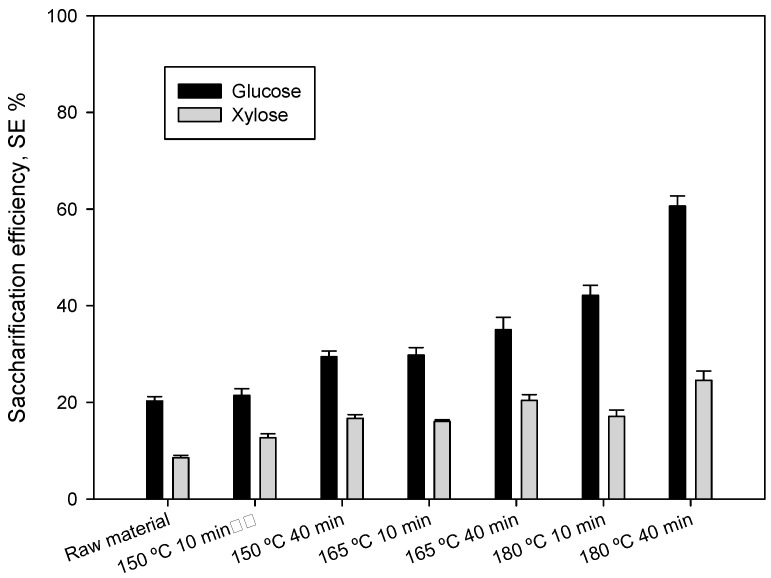
Saccharification efficiency (SE, %) of enzymatic hydrolysis performed with raw material and pretreated materials at different operating conditions. Data were calculated for total potential glucose and xylose.

The accessibility of cellulose from the pretreated material to enzymatic attack is one of the most important variables to measure the effectiveness of liquid hot water pretreatment [78]. The results of the enzymatic hydrolysis were expressed in terms of “saccharification efficiency” in Figure 4. Saccharification efficiency was calculated according to the procedure described in Section 2.6. Data were calculated for total potential glucose and xylose, not only from cellulose and hemicellulose. In the conditions tested and focused on glucose, the percentages of SE were between 21.44% (150 °C and 10 min) and 60.62% (180 °C and 40 min). The comparison of these results with that obtained on the raw material without pretreatment (20.3%) indicates that the pretreatment considerably improves the accessibility of enzymes to the pretreated material for breaking the complex polysaccharides, especially if pretreatment is performed at the highest temperature and residence time. Similar results have been reported by other researchers in their published studies [39,50]. Regarding the effect of residence time in the SE, at 150 °C, values changed from 21.44% (10 min) to 29.44% (40 min). At temperatures of 165 °C and 180 °C, a similar effect was observed, with values that increased from 29.79% and 42.14% (10 min) to 35.03% and 60.62% (40 min), respectively.

### 3.4. Global Efficiency of a Two-step Process of Pretreatment and Consecutive Enzymatic Hydrolysis

Table 2 shows a comparison between proposed process based on only one-step enzymatic hydrolysis and two-step pretreatment (in the different experimental conditions) and consecutive enzymatic hydrolysis. The severity factor (R_0_) indicates the severity of the pretreatment [79,80,81]. The equation to determine the severity factor that involves the reaction time and temperature is the following:

(7) R0=t×e(T−Tref)14.75

where *t* is the residence time (min); *T* is the temperature (°C); and *T*_ref_ is the reference temperature, usually set to 100 °C [82].

The global efficiency values were very similar to the saccharification efficiency in many of the tested conditions according to the glucose recovery in IS analysis, where low solubilization of this component in the liquid fraction after pretreatment was found. 

Regarding the overall glucose efficiency, experimental values obtained ranged between 17.20% and 61.02% of the potential glucose of the raw material. With regard to the 61.02% value, the majority of glucose came from the enzymatic hydrolysis stage, since during the pretreatment, only 14% of the glucose contained in the raw material was solubilized in the liquid fraction. From the data obtained, it can be concluded that, in order to reach high global glucose efficiency, a temperature of 180 °C and a medium residence time of 40 min should be chosen.

The global efficiency of xylose followed a similar pattern to that of pretreatment (xylose recovery evaluated in the liquid fraction), due to the low amount of xylose released in the enzymatic hydrolysis step. The highest efficiency was obtained also at 180 °C and 40 min of residence time, as for glucose. The global efficiency ranged from 8.58% for enzymatic hydrolysis without any previous pretreatment to 26.12% for a two-step process including a liquid hot water pretreatment at 180 °C for 40 min and an enzymatic hydrolysis. 

The above results of global efficiency of a two-step process involving pretreatment and consecutive enzymatic hydrolysis are reasonable for the sugar yield (60–70%), according to Kumar et al. [83]. Generally, our global efficiencies are in the (slightly lower) range of other studies found in the literature that were performed with different biomass materials. For example, Imman et al. [84] reported 82% of glucose yield for corncobs pretreated at 160 °C for 10 min. Pérez et al. [37] found 38% of xylose yield for wheat straw pretreated at 188 °C for 40 min. 

A correlation between the severity factor and glucose and xylose global efficiencies was not observed. A larger influence of temperature than time of pretreatment seems to occur.

The possible formation of fermentation inhibitors, particularly phenolic compounds, was investigated by analyzing the liquid fractions. Table 2 also reports the values of total polyphenol content in the different liquid fractions (only the liquid fractions obtained from the enzymatic hydrolysis of solid samples subjected or not to liquid hot water pretreatment). Liquid hot water pretreatment has been mainly used to extract phenolic compounds from many different sources, such as plants and food-industry byproducts [85,86,87,88,89]. In many of these studies, the total phenolic compounds and the total antioxidant capacity were measured. A higher total phenolic compound concentration and a higher antioxidant activity were observed in the extracts obtained at higher temperatures and at longer extraction times. This could explain that, when the pretreatment was performed, the phenolic compounds were released, and then, in the liquid fraction derived from enzymatic hydrolysis, they are in a lower concentration.

Particularly, phenolic compound concentrations are quite below concentrations that inhibit sugar fermentation. This agrees with the review performed by Jönsson and Martín [40] on pretreatment of lignocellulose. These authors reported that hydrothermal processing minimized the formation of fermentation inhibitors and particularly phenolic compounds. With regard to concentrations to get an inhibitory impact, Fosso-Kankeu et al. [90] found that the inhibitory effect of phenols on the viability of *Saccharomyces cerevisiae*, and therefore on ethanol productivity, was not significant at concentrations of 2000 mg/L of vanillin. Yeast was able to adapt to a medium with this concentration of vanillin. Instead, they found total inhibition of bioethanol production when working with a vanillin concentration of 4000 mg/L. In addition, Allard-Massicotte et al. [91], in their study about phenols removal from pre-hydrolysate by laccase, reported that a phenolic compounds concentration of 280 mg/L was far below the limit of inhibition in the acetone-butanol-ethanol fermentation.

Finally, Table 3 shows the analysis of variance, two-way ANOVA test, used to simultaneously evaluate the effect of time and temperature on global efficiency (glucose and xylose, %) and phenolic compounds concentration. The results indicated that the global efficiency in the range studied was significantly (*p* < 0.05) affected by the two factors, time and temperature. However, there was no difference in the means of both factors, temperature and time, when phenolic compounds concentration was evaluated. Also, there is no indication of important differences in the data of different blocks or replicates at the 5.0% significance level.

## 4. Conclusions

Liquid hot water pretreatment is a promising and environmentally friendly procedure to improve enzymatic hydrolysis of a very common vegetable waste, Italian green pepper, to produce fermentable sugars. An increase in temperature and contact time during pretreatment produced a positive effect on glucose recovery. Particularly, the incorporation of the liquid hot water step at 180 °C for 40 min before enzymatic hydrolysis resulted in a three-fold increase in glucose (from 20.3% to 61.0%) and xylose (from 8.6% to 26.1%) yields. Concentrations of phenolic compounds were very low to inhibit later sugar fermentation.

Numerous researchers are currently working with lignocellulosic biomass, especially agricultural byproducts and wastes, because they are abundant, inexpensive, sustainable, and biodegradable. Italian green pepper is very commonly used in Mediterranean cooking, and, as such, waste levels of this food are significantly high. We believe that the enormous quantity of this food being discarded annually should encourage looking into alternative treatments to maximize the value of this interesting feedstock.

## Figures and Tables

**Table 1:** Main composition of green pepper used as raw material in this work (data in g by 100 g of dry weight).

Analysis		Data
Structural and nutritional analysis	Carbohydrates (data adapted from USDA)	75.9
	Protein (data adapted from USDA)	14.1
	Total lipid (data adapted from USDA)	2.8
	Lignin	8.7 ± 0.8
	Cellulose	24.8 ± 1.9
	Hemicellulose	10.7 ± 1.1
	Extractives	2.3 ± 0.1
Proximate analysis	Equilibrium moisture	3.0 ± 0.1
	Fixed carbon	14.7 ± 1.9
	Volatile matter	75.3 ± 3.6
	Ashes	7.0 ± 0.4
Elemental analysis	Carbon, C%	43.9 ± 1.2
	Nitrogen, N%	3.2 ± 0.2
	Hydrogen, H%	6.4 ± 0.4
	Sulfur, S%	<0.1
	Oxygen, O%	39.4 ± 1.0
Potential sugars (experimental data)	Glucose	33.7 ± 3.4
	Xylose	11.4 ± 0.7
	Fructose	0.9 ± 0.1
	Lactose	3.4 ± 0.4
	Sacarose	1.1 ± 0.3

**Table 2:** Global efficiency of one- and two-step hydrolyses (data were calculated for total glucose and xylose) and phenolic compounds concentration on liquid fractions.

	Process						
	No Pretreatment	150 °C		165 °C		180 °C	
		10 min	40 min	10 min	40 min	10 min	40 min
Log R_0_	-	2.5	2.9	3.4	3.1	3.5	4.0
Glucose, %	20.30 ± 1.08 ^B^	17.20 ± 1.68 ^A^	27.86 ± 0.76 ^D^	24.75 ± 2.56 ^C^	45.84 ± 0.90 ^F^	34.56 ± 0.52 ^E^	61.02 ± 1.75 ^G^
Xylose, %	8.58 ± 0.42 ^A^	8.60 ± 0.51 ^A^	13.94 ± 1.49 ^B^	12.89 ± 0.91 ^B^	19.03 ± 0.76 ^C^	13.64 ± 0.50 ^B^	26.12 ± 1.01 ^D^
Phenolic compounds concentration, mg/L	605.57 ± 29.40 ^E^	72.24 ± 6.51 ^AB^	80.36 ± 3.16 ^D^	79.81 ± 6.24 ^D^	77.44 ± 9.00 ^CD^	75.58 ± 2.05 ^BC^	69.12 ± 9.41 ^A^

^A–G^ Means with different letters in the same row showed statistically significant differences at the 95% confidence level. Reader can see more details in Tables S1–S6 of Supplementary Material.

**Table 3:** Analysis of variance (ANOVA) for the comparison of differences between means.

Dependent Variable	Source	Type III Sum of Squares	Df	Mean Square	F-Ratio	*p*-Value
**Glucose, %**	**Main effects**					
	Temperature	1914.2	1	1914.2	126.89	0.0000
	Time	1694.4	1	1694.4	112.32	0.0000
	Blocks-replicates	5.77951	2	2.88976	0.19	0.8280
	**Total error**	196.115	13	15.0858		
	**Total (corrected)**	3810.28	17			
**Xylose, %**	**Main effects**					
	Temperature	222.396	1	222.396	57.92	0.0000
	Time	287.041	1	287.041	78.65	0.0000
	Blocks-replicates	1.77303	2	0.886517	0.23	0.7970
	**Total error**	49.9145	13	3.83957		
	**Total (corrected)**	561.125	17			
**Phenolic compounds concentration, mg/L**	**Main effects**					
	Temperature	59.4075	1	59.4075	3.34	0.0905
	Time	1.46205	1	1.46205	0.08	0.7787
	Blocks-replicates	30.4436	2	15.2218	0.86	0.4471
	**Total error**	230.943	13	17.7649		
	**Total (corrected)**	327.256	17			

## Supplementary Materials

The following are available online at https://www.mdpi.com/2304-8158/9/11/1640/s1.
