# Supplementary material for: Liquid Hot Water Pretreatment and Enzymatic Hydrolysis as a Valorization Route of Italian Green Pepper Waste to Delivery Free Sugars
Source: Foods. 2020 Nov 10;9(11):1640. doi: 10.3390/foods9111640 (PMC7697518; doi:10.3390/foods9111640)
Supplement: Supplementary file 1 [file foods-09-01640-s001.pdf]

SUPPLEMENTARY MATERIAL

Liquid Hot Water Pretreatment and Enzymatic Hydrolysis as a Valorization Route of Italian Green Pepper Waste to Delivery Free Sugars

M.A. Martín-Lara [1,*], L. Chica-Redecillas [1], A. Pérez [1], G. Blázquez [1], G. Garcia-Garcia [2] and M. Calero [1]

[1] Chemical Engineering Department. Faculty of Sciences. University of Granada. Avda. Fuentenueva, s/n, 18071 Granada (Spain)

[2] Department of Chemical and Biological Engineering. The University of Sheffield. Sir Robert Hadfield Building, Sheffield, S1 3JD (UK)

* Correspondence: marianml@ugr.es; Tel.: +34 958240445 (M.A.M.-L.)

Tables S1, S2, S3, S4, S5 and S6 apply a multiple comparison procedure to determine which means are significantly different from which others. Although there are several multiple comparison procedures, the Fisher's least significant difference (LSD) procedure has been chosen in this work. In Tables S1, S2 and S3, within each column, the levels containing X's form a group of means within which there are no statistically significant differences. With this method, there is a 5.0% risk of calling each pair of means significantly different when the actual difference equals 0. Tables S4, S5 and S6 show the estimated difference between each pair of means. An asterisk has been placed to indicate that these pairs show statistically significant differences at the 95.0% confidence level.

Table 1. Multiple range tests for glucose % by temperature, time and pre-treatment (Method 95.0 percent LSD).

			Count	LS Mean	LS Sigma	Homogeneous Groups
Glucose, %	Temperature	No pre-treatment	3	20.3	6.09899	X
		150 ºC	6	22.53	4.31264	X
		165 ºC	6	35.2967	4.31264	XX
		180 ºC	6	47.79	4.31264	X
	Time	No pre-treatment	3	20.3	6.26152	X
		10 min	9	25.5033	3.61509	X
		40 min	9	44.9067	3.61509	X
	Pre-treatment	150 ºC, 10 min	3	17.2	0.4693	X
		No pre-treatment	3	20.3	0.4693	X
		165 ºC, 10 min	3	24.75	0.4693	X
		150 ºC, 40 min	3	27.86	0.4693	X
		180 ºC, 10 min	3	34.56	0.4693	X
		165 ºC, 40 min	3	45.84	0.4693	X
		180 ºC, 40 min	3	61.02	0.4693	X

Table S1. Multiple range tests for xylose % and phenolic compounds concentration, mg/L by temperature, time and pre-treatment (Method 95.0 percent LSD).

			**Count**	**LS Mean**	**LS Sigma**	**Homogeneous Groups**
Xylose, %	Temperature	No pre-treatment	3	8.58	2.57651	X
		150 ºC	6	11.27	1.82187	XX
		165 ºC	6	15.96	1.82187	XX
		180 ºC	6	19.88	1.82187	X
	Time	No pre-treatment	3	8.58	2.25465	X
		10 min	9	11.71	1.30172	X
		40 min	9	19.6967	1.30172	X
	Pre-treatment	No pre-treatment	3	8.58	0.365481	X
		150 ºC, 10 min	3	8.6	0.365481	X
		165 ºC, 10 min	3	12.89	0.365481	X
		180 ºC, 10 min	3	13.64	0.365481	X
		150 ºC, 40 min	3	13.94	0.365481	X
		165 ºC, 40 min	3	19.03	0.365481	X
		180 ºC, 40 min	3	26.12	0.365481	X

Table S3. Multiple range tests for phenolic compounds concentration, mg/L by temperature, time and pre-treatment (Method 95.0 percent LSD).

			**Count**	**LS Mean**	**LS Sigma**	**Homogeneous Groups**
Phenolic compound concentration, mg/L	Temperature	150 ºC	6	76.3	1.35515	X
		180 ºC	6	77.97	1.35515	X
		165 ºC	6	80.085	1.35515	X
		No pre-treatment	3	605.57	1.91647	X
	Time	10 min	9	75.8767	0.929761	X
		40 min	9	80.36	0.929761	X
		No pre-treatment	3	605.57	1.61039	X
	Pre-treatment	180 ºC, 40 min	3	69.12	1.25141	X
		150 ºC, 10 min	3	72.24	1.25141	XX
		180 ºC, 10 min	3	75.58	1.25141	XX
		165 ºC, 40 min	3	77.44	1.25141	XX
		165 ºC, 10 min	3	79.81	1.25141	X
		150 ºC, 40 min	3	80.36	1.25141	X
		No pre-treatment	3	605.57	1.25141	X

Table S4. Contrasts for glucose % by temperature, time and pre-treatment (Method 95.0 percent LSD).

		Contrast	Sig.	Difference	+/- Limits
Glucose, %	Temperature	No pre-treatment – 150 ºC		-2.23	15.7597
		No pre-treatment – 165 ºC		-14.995	15.7597
		No pre-treatment – 180 ºC	*	-27.49	15.7597
		150 ºC – 165 ºC		-12.7667	12.8678
		150 ºC – 180 ºC	*	-25.26	12.8678
		165 ºC – 180 ºC		-12.4933	12.8678
	Time	No pre-treatment – 10 min		-5.20333	15.1901
		No pre-treatment – 40 min	*	-24.6067	15.1901
		10 min – 40 min	*	-19.4044	10.741
	Pre-treatment	No pre-treatment – 150 ºC, 10 min	*	3.1	1.42348
		No pre-treatment – 150 ºC, 40 min	*	-7.56	1.42348
		No pre-treatment – 165 ºC, 10 min	*	-4.45	1.42348
		No pre-treatment – 165 ºC, 40 min	*	-25.54	1.42348
		No pre-treatment – 180 ºC, 10 min	*	-14.26	1.42348
		No pre-treatment – 180 ºC, 40 min	*	-40.72	1.42348
		150 ºC, 10 min – 150 ºC, 40 min	*	-10.66	1.42348
		150 ºC, 10 min – 165 ºC, 10 min	*	-7.55	1.42348
		150 ºC, 10 min – 165 ºC, 40 min	*	-28.64	1.42348
		150 ºC, 10 min – 180 ºC, 10 min	*	-17.36	1.42348
		150 ºC, 10 min – 180 ºC, 40 min	*	-43.82	1.42348
		150 ºC, 40 min – 165 ºC, 10 min	*	3.11	1.42348
		150 ºC, 40 min – 165 ºC, 40 min	*	-17.98	1.42348
		150 ºC, 40 min – 180 ºC, 10 min	*	-6.7	1.42348
		150 ºC, 40 min – 180 ºC, 40 min	*	-33.16	1.42348
		165 ºC, 10 min – 165 ºC, 40 min	*	-21.09	1.42348
		165 ºC, 10 min – 180 ºC, 10 min	*	-9.81	1.42348
		165 ºC, 10 min – 180 ºC, 40 min	*	-36.27	1.42348
		165 ºC, 40 min – 180 ºC, 10 min	*	11.28	1.42348
		165 ºC, 40 min – 180 ºC, 40 min	*	-15.18	1.42348
		180 ºC, 10 min – 180 ºC, 40 min	*	-26.46	1.42348

Table S5. Contrasts for xylose % by temperature, time and pre-treatment (Method 95.0 percent LSD).

		Contrast	Sig.	Difference	+/- Limits
Xylose, %	Temperature	No pre-treatment – 150 ºC		-2.69	6.65768
		No pre-treatment – 165 ºC	*	-7.38	6.65768
		No pre-treatment – 180 ºC	*	-11.3	6.65768
		150 ºC – 165 ºC		-4.69	5.43597
		150 ºC – 180 ºC	*	-8.61	5.43597
		165 ºC – 180 ºC		-3.92	5.43597
	Time	No pre-treatment – 10 min		-3.13	5.46966
		No pre-treatment – 40 min	*	-11.1167	5.46966
		10 min – 40 min	*	-7.98667	3.86763
	Pre-treatment	No pre-treatment – 150 ºC, 10 min		-0.02	1.10857
		No pre-treatment – 150 ºC, 40 min	*	-5.36	1.10857
		No pre-treatment – 165 ºC, 10 min	*	-4.31	1.10857
		No pre-treatment – 165 ºC, 40 min	*	-10.45	1.10857
		No pre-treatment – 180 ºC, 10 min	*	-5.06	1.10857
		No pre-treatment – 180 ºC, 40 min	*	-17.54	1.10857
		150 ºC, 10 min – 150 ºC, 40 min	*	-5.34	1.10857
		150 ºC, 10 min – 165 ºC, 10 min	*	-4.29	1.10857

	150 ºC, 10 min – 165 ºC, 40 min	*	-10.43	1.10857
	150 ºC, 10 min – 180 ºC, 10 min	*	-5.04	1.10857
	150 ºC, 10 min – 180 ºC, 40 min	*	-17.52	1.10857
	150 ºC, 40 min – 165 ºC, 10 min		1.05	1.10857
	150 ºC, 40 min – 165 ºC, 40 min	*	-5.09	1.10857
	150 ºC, 40 min – 180 ºC, 10 min		0.3	1.10857
	150 ºC, 40 min – 180 ºC, 40 min	*	-12.18	1.10857
	165 ºC, 10 min – 165 ºC, 40 min	*	-6.14	1.10857
	165 ºC, 10 min – 180 ºC, 10 min		-0.75	1.10857
	165 ºC, 10 min – 180 ºC, 40 min	*	-13.23	1.10857
	165 ºC, 40 min – 180 ºC, 10 min	*	5.39	1.10857
	165 ºC, 40 min – 180 ºC, 40 min	*	-7.09	1.10857
	180 ºC, 10 min – 180 ºC, 40 min	*	-12.48	1.10857

Table S6. Contrasts for phenolic compounds concentration, mg/L by temperature, time and pre-treatment (Method 95.0 percent LSD).

		**Contrast**	**Sig.**	**Difference**	**+/- Limits**
Phenolic compounds concentration, mg/L	Temperature	No pre-treatment – 150 ºC	*	529.27	4.95215
		No pre-treatment – 165 ºC	*	525.485	4.95215
		No pre-treatment – 180 ºC	*	527.6	4.95215
		150 ºC – 165 ºC		-3.785	4.04341
		150 ºC – 180 ºC		-1.67	4.04341
		165 ºC – 180 ºC		2.115	4.04341
	Time	No pre-treatment – 10 min	*	529.693	3.90672
		No pre-treatment – 40 min	*	525.21	3.90672
		10 min – 40 min	*	-4.48333	2.76247
	Pre-treatment	No pre-treatment – 150 ºC, 10 min	*	533.33	3.79576
		No pre-treatment – 150 ºC, 40 min	*	525.21	3.79576
		No pre-treatment – 165 ºC, 10 min	*	525.76	3.79576
		No pre-treatment – 165 ºC, 40 min	*	528.13	3.79576
		No pre-treatment – 180 ºC, 10 min	*	529.99	3.79576
		No pre-treatment – 180 ºC, 40 min	*	536.45	3.79576
		150 ºC, 10 min – 150 ºC, 40 min	*	-8.12	3.79576
		150 ºC, 10 min – 165 ºC, 10 min	*	-7.57	3.79576
		150 ºC, 10 min – 165 ºC, 40 min	*	-5.2	3.79576
		150 ºC, 10 min – 180 ºC, 10 min		-3.34	3.79576
		150 ºC, 10 min – 180 ºC, 40 min		3.12	3.79576
		150 ºC, 40 min – 165 ºC, 10 min		0.55	3.79576
		150 ºC, 40 min – 165 ºC, 40 min		2.92	3.79576
		150 ºC, 40 min – 180 ºC, 10 min	*	4.78	3.79576
		150 ºC, 40 min – 180 ºC, 40 min	*	11.24	3.79576
		165 ºC, 10 min – 165 ºC, 40 min		2.37	3.79576
		165 ºC, 10 min – 180 ºC, 10 min	*	4.23	3.79576
		165 ºC, 10 min – 180 ºC, 40 min	*	10.69	3.79576
		165 ºC, 40 min – 180 ºC, 10 min		1.86	3.79576
		165 ºC, 40 min – 180 ºC, 40 min	*	8.32	3.79576
		180 ºC, 10 min – 180 ºC, 40 min	*	6.46	3.79576
